# Volatile Fragility: New Employment Forms and Disrupted Employment Protection in the New Economy

**DOI:** 10.3390/ijerph17051531

**Published:** 2020-02-27

**Authors:** Bin Chen, Tao Liu, Yingqi Wang

**Affiliations:** 1 Geschwister Scholl Institute of Political Science, Ludwig Maximilian University of Munich, 80538 Munich, Germany; bin.chen.lmu@gmail.com; 2 School of Public Affairs, Zhejiang University, Hangzhou 310007, China; 3 Institute of East Asian Studies, University Duisburg-Essen, 47057 Duisburg, Germany; 4 Institute for Sociology, University Duisburg-Essen, 47057 Duisburg, Germany; 5 Center for Social Security Studies, Wuhan University, Wuhan 430072, China

**Keywords:** digital age, employment, labor, protection, volatility, fragility

## Abstract

This research is based on empirical surveys conducted in two Chinese cities, Beijing and Chengdu, which examine employment relationships, labor protection and social protection in the new digital economy. Through these theoretically informed surveys on various forms of employment via online platforms, we have found that the organizational principles and functional patterns of employment have profoundly transformed in the epoch of digitalization. The traditional employment relationship characterized by written contracts with clearly defined entitlements and obligations for employers and employees have been increasingly substituted by new volatile, fluid and fragile employment forms, softening the labor rights and social rights of “digital employees” and strengthening social control over them through online evaluation systems supported by smart phones and apps. The employees engaged in the online sharing economy have become more individualized and atomized than ever before, resulting in the emergence of an unorganized and disenfranchised “digital working class”.

## 1. Introduction

Conventionally, modern labor protection systems corresponding to the material economy are jointly established by governments, employers and unions. Existing frameworks for employment legislation and social policies created by governments (such as minimum wages, paid holidays and sick leave, occupation safety and health) are built around and delivered through traditionally organized business firms [1]. A definitive and relatively consistent employment relationship relying on a labor contract is the premise for employees to obtain the above-named labor protections. Pensions, health insurance, unemployment insurance, workers’ compensation and disability insurance are all linked to employment, provided as social benefits to workers holding a regular job [2]. In addition, labor unions are important organizations to safeguard the rights and interests of workers. The emergence of such comprehensive labor protection coincides with industrialization and the diffusion of the mass production methods of capitalism. In the post-war period, the expansion of various labor protection measures in many different regions has caused the rapid development of Western welfare states into an explosive development trajectory [3].

Faced with the emergence and rapid dissemination of an internet-based digital economy, employment forms and patterns have changed accordingly. There are no fixed working hours and workers are able to offer their activities through apps and platforms whenever they want. The digital economy, then, enables workers to benefit from job opportunities that they might not otherwise be able to obtain, and they can flexibly arrange working hours to complement the performance of other work, family-related, study, or leisure activities [4]. Recently, millions of workers have become involved in this new employment field. However, the advantages of flexibility may conceal the disadvantages of being deprived of essential labor protection rights. Platform companies usually do not consider themselves employers, but only platforms, networks or intermediaries, and their workers are formally treated as self-employed or freelancers. The classical employer-employee relationship is not transparent in the virtual economy; labor protection institutions (such as social insurance) based upon employment contracts and clearly delineated labor relations have been disrupted. Furthermore, the design and application of rating systems driven by clients can directly impact platform workers’ income or even access to future work. Digital employment can create a class of isolated individuals living from job to job, without social connections to workplaces or to other workers [2], making it difficult for digital workers to protect their rights through collective action or to establish a union. Consequently, platform workers are in an extremely vulnerable position in this new economic model. How can the rights and interests of platform workers be protected? How workers in the new digital economy can be secured and insured represents an urgent problem.

Taking China as an exemplary case to test the impact of the digital economy on labor protection is appropriate not only because it is the world’s second largest economy, but also because it has experienced rapid development of the digital economy over the last two decades, compared with other countries in the world. According to the White Paper on Development and Employment of China’s Digital Economy (2019), released by the China Academy of Information and Communications Technology (CAICT), the scale of China’s digital economy reached 31.3 trillion yuan ($4.48 trillion) in 2018, accounting for 34.8 percent of gross domestic product (GDP); people who worked in the digital economy numbered 191 million in 2018, accounting for 24.6 percent of total employment that year. However, the vast majority of platform workers are usually treated as self-employed or freelancers, who are excluded from labor protection systems that target only workers with a formal employment relationship. State agencies and policymakers will therefore face a significant challenge to bring labor protection mechanisms into line with new flexible employment relationships and to ensure social protection for all types of workers.

For this study, we have conducted empirical research in two Chinese cities to explore labor protection and social protection for Chinese platform workers engaged in the digital labor market. The remainder of the paper is organized as follows: Section 2 constructs a theoretical analysis framework. Section 3 introduces information about the methods and data we use for the analysis. Section 4 presents the research findings, including the concepts of the invisible employer, the strong client, atomized employees and ineffective state regulation. Section 5 offers our conclusions.

## 2. Analytical Framework

### 2.1. Theoretical Approaches to Employment Relationships and Labor Protection in Industrial Societies

The research field on employment relationships encompasses a repertoire of topics, concepts, scripts and norms that place emphasis on the relationship between employers and employees, the institutional setting in the industrial process, power distribution and configuration in factories, as well as mechanisms and arrangements for mediating interests and alleviating conflicts in the market economy. From a historical perspective, concerns about the employment relationship have appeared against the backdrop of the emergence and expansion of the capitalist mode of production, significantly shaped by the surge of criticism and reflection about the drawbacks and deficits of the capitalist system. The Marxist school of thought has concentrated on escalating conflicts between two primary classes, and the trend of pauperization and proletarianization of the working class, uncovering the enigma of additional values and exploitation of the labor force in the capitalist order [5,6]. Polanyi has proposed the concept of the “market society”, noting the trend toward a “disembedded economy”. In other words, the market economy has become disembedded from social relationships and social institutions, and the decoupling of economy from society has severely weakened social ties and social linkages, jeopardizing reciprocity and redistribution in capitalist society [7]. Over the course of the temporal and spatial expansion of the capitalist market economy, the labor force has been increasingly coerced into taking part in the industrial production process, and members of market societies have been exposed to the functional primacy and hegemony of commodification. 

By connecting the four dimensions of: (1) the labor process, (2) the mode of labor reproduction, (3) market competition and (4) state intervention, Burawoy has proposed the concept of “factory regimes”, typologizing this core concept into two subcategories: “despotic regimes” and “hegemonic regimes”. “Factory despotism” is linked to the early and more primitive period of capitalism, within which the primary methods of social control were coercive and suppressive in the labor process, assuming a despotic character. The market’s dominance over workers, the vast power gap between capital and labor, the rigorous factory discipline, the cutthroat job competition and the atomization of individual workers are the primary features of the factory despotic regimes [8]. The second type of regime, the hegemonic, has emerged in the course of constituting monopoly capitalism. The despotic character of factory regimes has declined, and the employment relationship is no longer characterized by direct social control and the use of suppressive methods. Instead, the employment relationship, along with the capital-labor relationship, have been preferentially shaped by non-coercive methods, including persuasion, cooperation and agreement, achieved through institutional mechanisms mediating interests. In hegemonic regimes, consent prevails in the employment relationship (ibid). The main impetus driving the transformation from despotic regimes to hegemonic regimes has been increased state intervention in the production and reproduction of the labor force. Emerging welfare states have intensively corrected the market society under the shadow of the coercion of commodification, intervening not only in the area of labor protection, such as policies regulating working time, remuneration, working conditions and working environments, but also in the area of social protection, establishing pension insurance, health insurance, unemployment insurance, maternity protection, etc. [8,9,10]. Accordingly, the legal status and position of industrial workers have been remarkably enhanced through ensuring social and labor rights for this empowered group [11].

Corresponding to the transitionary development of “factory regimes”, a few scholars have proposed the concept of “decommodification” to portray the trend away from the “commodification” of the early capitalist economy. “Decommodification” measures the degree of alleviation of the commodified character of the labor force and the empowerment of individual employees independent from the coercion of market mechanisms [12,13]. According to Esping-Andersen, decommodification has been strengthened through arrangements of modern welfare states, and a welfare state with a high degree of decommodification (like countries in the Scandinavian region) relieves those in the labor force from their heavy dependence upon the market and safeguards their material security and wellbeing in the case of their exit from the market owing to invalidity, aging, illness, unemployment, maternity leave or other reasons, such as occupational training [13]. Decommodification reflects Polyani’s prediction on the “double movement” in capitalist society. The penetration of the capitalist production process assumes the universal function of turning all goods, and even labor, into commodities. At the same time, society has resisted this trend of the universalization of market mechanisms and has endeavored to defend its independent terrain through the tenet of “self-protection of society” [7]. The trend of decommodification has mitigated the absolute power of market despotism and shaped a more balanced industrial relationship between capital and labor in favor of the latter, mitigating the power asymmetry between employers and employees. Core institutional arrangements facilitating a more balanced and sustainable employment relationship include the association of workers in the form of organizing trade unions, establishment of institutional mechanisms for mediating interests through negotiation, social partnership and tripartism, and the promotion of collective bargaining rights of working classes. Comprehensive labor and social protection and unequivocally entrenching of labor and social rights, such as the rights to labor contracts, paid holidays, rest periods, salaries, safe working environments, association, social insurance, continued remuneration in the case of sickness and maternity leave, etc. have further elevated the social and legal status of the labor force, constraining the arbitrary despotism of capital. In the employment relationship, the employment contract offers significant protection for workers’ social and labor rights, in an institutional and formal (written) form based on conditioning rules that are socially prescribed and sanctioned [14]. For instance, clauses concerning protection against dismissal represent an important arrangement for protecting labor rights and reducing market contingency.

### 2.2. Digital Challenges to the Employment Relationship and Labor Protection

Digitalization has on the one hand generated infinite possibilities for new employment forms and production modes transcending temporal and spatial constraints; on the other hand, digitalization has led human society into a period of unprecedented uncertainty. The employment relationship and the employment protections developed from the early capitalist period and the modern welfare state, which are embedded in a stable and reliable legal framework, encounter a new epoch of welfare intangibility and contingency that softens traditional labor laws and social laws [15]. Can these employment forms from industrial and post-industrial society survive in an accelerated, digitalizing society? Are traditional forms of labor protection and social protection compatible with various new digital employment forms? How have new forms of digital employment affected the traditional employment relationship, labor protection and social protection? Will decommodification decline in favor of a rising trend of re-commodification in a digital labor society? Through an empirical study about digital employment in China, we seek to provide further theoretical discussion and reflection regarding the changes in and challenges to the employment relationship in the course of digitalization. 

## 3. Methodology

This study is part of a project on labor protection for platform workers in the digital age, which applied a qualitative research approach. The core methods included semi-structured, in-depth interviews with platform workers (food delivery workers and ride-hailing drivers) and other platform work stakeholders (platform managers, policy-makers). Data collection was conducted in Beijing and Chengdu from July to September of 2018.

The two cities in different part of China were selected as research sites because of the extent of development in their e-economies and high rates of e-employment. In April 2018, the China Academy of Information and Communications Technology issued a White Paper on the Development and Employment of China’s Digital Economy; according to the paper, the scale of China’s digital economy reached 27.2 trillion yuan ($3.86 trillion) in 2017. Among all provinces, the digital economy in Beijing accounted for the highest proportion of GDP, reaching 49.7 percent [16]. Chengdu, located in the western part of China, is the most developed city in this generally peripheral region, in terms of both its offline and online economies. The two types of e-employees were chosen mainly based on the number of e-workers and the kinds of services they provide. According to the Report on the Development of the Sharing Economy in China, released by the Chinese State Information Center (SIC) in 2017, platform workers were concentrated in the area of living services (such as food delivery and courier services) and driving; in 2016, the former had a scale of 20 million workers and the latter numbered more than 18 million [17].

The data were collected by the authors during fieldwork in the two cities, and the primary method used to collect the data was semi-structured, in-depth interviews. Participants were recruited through listings on three online platforms (Eleme, Meituan for food delivery, DiDi for driving), meeting a range of predefined research criteria, including type of platform work, length of employment, working hours and location. A sample of 32 individuals was selected in Beijing and 25 people in Chengdu. In the end, 46 platform workers (24 for food delivery and 22 for ride-hailing) were interviewed in face-to-face settings, and participants answered open-ended questions covering socioeconomic background, working conditions, occupational risks, awareness of labor protection and other difficulties they may have encountered in their work. The majority of platform worker participants in Beijing were from other provinces; in Chengdu, however, it was the opposite. The specific characteristics of all participants are summarized in Table 1. Our primary criterion for ending the field interview process was whether “theoretical saturation” had been achieved [18]. When approximately 30 platform workers had been interviewed, it was found that the core themes and topics were being constantly repeated by the narrators, demonstrating that we had nearly reached the point of theoretical saturation. Our analysis was also informed by eight additional interviews with a range of platform work stakeholders (six with platform managers and two with policy-makers). All interviews lasted for an average of one hour each. Taking ethical considerations such as anonymity and confidentiality into account, all informants’ names have been anonymized with pseudonyms and participants were informed that only those working in our research project would have access to the data.

## 4. Findings

### 4.1. Invisible Employers: Platform Workers Are Unable to Receive Protection from Them

In line with the Annual Report on China’s Sharing Economy Development (2019), released by the Chinese State Information Center, the transaction scale of the sharing economy in China reached 2942 billion yuan ($419.8 billion) in 2018, about 75 million people were employed in this field and the number of employed persons is increasing. However, only 5.98 million people are actually employees of these platform companies, accounting for less than eight percent of the total number; the vast majority of platform workers are treated as self-employed or freelancers. In terms of the employment contract or the employment relationship stipulated by national laws, most platforms will deny having platform workers [19]. Only six out 46 interviewed platform workers had a labor contract (five for food delivery workers and one for ride-hailing drivers, see Table 1). Therefore, most of them do not fall under the purview of any of the protections typical for an employment relationship, such as paid holidays, a minimum wage, occupational safety and health, sick leave, unemployment, or retirement pension, to name a few [20,21].

There are two primary reasons that online platforms position workers as self-employed. The first is that platform companies generally consider themselves intermediaries and matchers of demand and labor supply; they do not employ the people providing services [21,22]. The other is that platform work features relatively high autonomy in terms of when, where and how work is performed. Platform workers can work simultaneously on several platforms or even have another job that classifies them as employees, and doing platform work simply increases their income [4,19]. As one of the platform managers described:

“Platform workers have great autonomy in terms of work time, workplace and the type of work. Our platform is only responsible for the development and operation of application software and service information promotion; we do not directly operate any physical business or employ people to provide specific services. Therefore, we have no obligation to take on employer responsibilities as specified in the employment relationship”.

However, platforms often act as more than simply intermediaries. They may “hide” their employer roles, acting as “invisible” employers. The key issue, with important implications for the provision of labor protections for platform workers, concerns the level of control exercised by platforms over the conduct of work, and vice versa, the degree to which workers exercise autonomous control over their work. This is a critical issue because control is an important element in the legal classification of an employee, and the legal status of employee (or worker) is the gateway to many important social protections [23]. In reality, some new control methods have been implemented in the platform economy, and a considerable number of workers actually risk being in a disguised employment relationship, rather than genuinely “free” [21], for the following reasons.

First, the platform can implement real-time monitoring of workers through various technical means; namely, they can exercise “digital control”. The example of ride-hailing drivers offers a good illustration for this type of control. Even though these drivers can enjoy a relatively high degree of autonomy in their work, geolocation devices are in a position to manage their route planning or locations. Meanwhile, their driving times, working hours, and even what they have communicated with clients in their cars are automatically recorded by such devices [24,25]. As one driver explained, he felt he didn’t have much autonomy in platform work because he was always under observation and “policed” by the app in real time, and he would be punished for saying something wrong to customers or parking in an unspecified location.

Second, each platform establishes its own regulations with which workers must comply, such as those granting rights to create accounts or paying commissions. For instance, DiDi drivers must pay an average commission of 19 percent per order, and some orders are even higher than 25 percent [26]. At the same time, the platforms are usually free to modify these rules unilaterally at any time, as well to deactivate worker accounts [27,28]. Most of the platforms explicitly specify in their terms and conditions that workers must communicate with clients through the platform; otherwise, their accounts will be blocked if they attempt to work directly with clients. Furthermore, platform workers have no power to negotiate the prices set by the platform. In other words, if platform workers are really independent contractors, they should have the right to directly communicate with clients and negotiate prices by themselves. Instead, the platforms exercise monopolistic power: in order to get a job, platform workers must abide by the instructions and rules unilaterally set by the platforms or apps.

Third, the rating and incentive systems, in essence, also represent forms of control. The rating systems used in virtually all platform businesses to establish trust between workers and clients directly determine worker revenue or even future work [25]. Moreover, such systems can automatically “recognize” performance that is poor or perceived to be as such, offering a way to implement control [4]. Meanwhile, low ratings can even constitute a sole reason for workers to be “fired” by the platform. Therefore, in order to achieve and maintain high ratings and ensure future work, a platform worker may work in an excessive burst to complete the task within the agreed time or sooner. Furthermore, to improve their operational efficiency, some platforms encourage workers to work longer hours through offering various incentives, and accounts may be closed down due to long-term inactivity. As one informant explained:

“*In the take-away industry, we are most afraid of being poorly rated by clients. A bad review means one day of useless work (at least 200 yuan is deducted). If there are three bad reviews within a month, then my account will be banned from the platform. According to the rules set by the platform, I can only get rewards by sending 40 orders in one day. Therefore, in order to get rewards and avoid bad reviews, I can only extend my working time, or violate traffic regulations to strive for more time; there is no other way*”.

### 4.2. Strong Clients: Platform Workers Cannot Obtain Equal Rights and Protections 

One of key features of the platform economy consists of the trust mechanisms between platform workers and clients established by the rating systems commonly used by platform businesses. Ratings by clients can directly affect the interests of service providers, especially their revenue or even access to future work on a particular platform. As a new dominant “medium”, the rating system leads to extremely unequal relations between workers and clients, posing risks to the physical and psychological well-being of platform workers in several ways.

First, the platform worker’s job or income is more precarious because of client-led rating systems. As mentioned above, the digital rating systems used in many forms of platform employment may determine not only if workers are able to obtain a reasonable income, but also whether they can continue working on the particular app at all. Platform workers that can be “summoned” by clients clicking their mouse or taping their mobile phone often complete the task and then simply disappear into the crowd or into the on-demand workforce. They are expected to run as smoothly as the software or a technological tool. However, if something goes wrong, they may receive worse comments or feedback than their counterparts in other economic sectors. This, in turn, may have a serious impact on their ability to work or earn in the future, because whether platform workers can continue to use a specific app or obtain more remunerable jobs on platforms depends heavily on the ratings and reviews of their past activities [4]. Bad reviews from clients may result in a sharp decline in one’s ranking, which would prevent a platform worker from getting the higher-paying jobs available only to workers with the highest rankings. Furthermore, some clients may refuse to pay, affecting the platform worker’s income. As one informant described, he once met a drunken passenger, and drove him to the place designated in the app; however, the location the passenger had initially entered into the app was wrong. After exiting the car, the passenger not only refused to pay the fee, but also gave the driver a bad review, and the driver could not find any way to make up for his loss.

Second, platform workers face many physical hazards due to client-oriented rating systems. In order to avoid bad ratings, workers must respond to client needs rapidly. For example, once a client places an order, the worker must complete the task as soon as possible, in order to achieve high ratings and ensure future work, or even to receive payment for the task itself. As a result, platform workers may become overworked, without taking enough breaks or nighttime rest [19]. Further, as one food delivery worker described, platform workers often violate traffic rules (such as retrogradation, or running a red light):

“*In fact, I know that I should abide by the traffic rules, but the delivery time specified by the platform is getting shorter and shorter. In order not to get bad comments from customers and lead to a loss in revenue, sometimes we must run the red light. You just say to yourself, ‘Who is not afraid of death?*’”

Among ride-hailing drivers, who must pay attention to the messages or phone calls sent by passengers via apps, such distractions can lead to serious traffic accidents. In short, whether working long hours, violating traffic regulations or risking accidents, working on the platform is characterized by a number of physical risks for workers through rating systems.

In addition, there exist psychological risks related to client-driven rating systems. For one, the intensity of work, which encourages a rapid pace without breaks, can contribute to psychosocial disorders. In order to get good reviews or ensure future work, the platform worker is under pressure to meet the specified timeframe or tight deadlines. Moreover, the rating systems are often regarded as a key source of worry for workers, who sometimes feel punished by “revenge” ratings or discriminatory ratings, which are outside of their control. As one informant described:

“*Due to the inaccurate location given by the app, I spent a long time finding one passenger. Instead of complaining about the app, however, she attributed the responsibility to me and gave me a poor review, but this was not my fault, I have to bear the loss, it was unfair. […] The platform would not give me a chance to explain; therefore, sometimes I feel helpless*”.

Finally, the need to perform emotional labor for clients can be mentally exhausting. Platform work that is carried out in the physical world may require workers to offer freebies (such as water or candy in the case of ride-hailing drivers), be exceptionally cheerful or even tolerate inappropriate behavior from clients [29,30], which also brings psychological risks to workers. As one informant said:

“*I had experienced several times when passengers took off their shoes in my car and spilled drinks on the seat, but I did not dare to complain to them, for fear of being badly rated by these passengers. However, I was actually angry in my heart. In fact, I always feel that the rating system is very unfair to us*”.

### 4.3. “Atomized” Laborers: Platform Workers Cannot Share Risks with Each Other

As illustrated above, since the platforms outsource their employer responsibilities, clients can directly have an impact on workers’ income, or even their future work, through rating systems. Platform workers are thus in the weakest position of the triangular relationship. However, for several reasons, they can’t share the risks associated with platform work with each other and it is difficult to organize to protect their own labor rights.

First, platform businesses disaggregate the workplace, both geographically and socially, creating a class of isolated individuals who never have a chance to connect with each other [2,31]. In the case of the platform economy, workers are generally treated as self-employed and they don’t have a common workplace. The majority of the tasks are performed individually, separated from any colleagues. The precarious position of platform workers, along with high turn-over rates, can lead to isolation and hinder the establishment of collective organizations or interest groups that could protect their rights and interests. Having no access to collective bargaining and worker interest groups [32], which means that they are forced to sue platforms or employers when problems arise, can be very expensive [33]. As one informant explained:

“*In fact, it’s quite lonely and sometimes helpless to do this kind of platform work. For example, once I had an accident on the way to deliver food. I was afraid to ask for insurance reimbursement (although the platform had purchased accident insurance for us) because the account may be cancelled by the platform company for seeking reimbursement. Finally, I chose to solve it privately. […] I don’t know any organizations or unions that I can turn to for discussing my work concerns or seeking help when faced with these kinds of problems*”.

Second, platform businesses, using inter-worker competition and rating systems, encourage workers to directly compete with one another, which is even less conducive to the solidarity and collaboration needed for effective unionization. Many of the workers simply don’t know each other, and, moreover, the individual ratings and competitive methods of work allocation designed by the platforms always remind workers that they are supposed to compete with each other. They can be easily replaced if they don’t “grab” at the clients’ orders or withdraw their labor. In such a situation, despite the many challenges faced by platform workers, some of them may not have any plans to strengthen their position through collective action or acts of solidarity [34]. One informant noted that he was not aware of any forms of cooperation among platform workers. They are replaceable because the threshold to entry is low; someone could quickly seize the order or the job provided by the clients. Therefore, he thought they (platform workers) were more like competitors rather than colleagues.

Finally, platform workers are generally younger, and they do not pay enough attention to labor protection issues. Workers in the platform economy tend to be younger than average, although some older and retired individuals also participate. The average age of our informants is 33.4, more than 70 percent less than 35 years old (see Table 1). Because they are still relatively young, they feel they don’t need to take part in social insurance or attach importance to other labor protection policies; it is better to put the money into their pockets:

“*Now I am still young. It is no problem for my employer, who doesn’t want to buy social insurance for me. I was going to think about it a few years later, so it would be better if my employer exchanges the potential contributions to social security into cash and adds to my salary*”.

### 4.4. Ineffective State Regulation: Platform Workers Are Excluded from Social Protection Policies

Platform workers cannot be protected by platforms because they are categorized as self-employed. Meanwhile, the stability of their income and occupation is directly affected by client-led rating systems. Because they are isolated from each other, it is extremely difficult to organize to protect their rights and interests, especially in areas such as occupational safety and health. However, even though workers in the platform economy are in such a weak position, the government has not played an effective role in protecting them; due to new regulatory loopholes in digital spaces [35], a large number of platform workers have been excluded from existing labor protection policies.

First, the existing framework of employment legislation and many social protection policies are only accessible through a standard model of employment, not designed for those construed as self-employed. For example, according to the “Social Insurance Law of the People’s Republic of China”, in addition to the basic endowment insurance and basic medical insurance that can be obtained by flexible employees, other protections, such as employment injury insurance, unemployment insurance and maternity insurance are either paid jointly by employers and employees or by separate payment from the employer. The premise for enjoying these social insurance benefits is that the employee and employer sign a formal labor contract, which forms a legal labor relationship. However, platform workers are more likely to be regarded by platforms as self-employed contractors rather than employees or workers, as they are considered to have a relatively high autonomy in terms of working time and place. Consequently, the standard employment model is absent, and these workers find themselves outside of a range of social protections. If they purchase social insurance themselves, the cost is much higher than paying through an employer. Although the average net income last month reached 7720.5 yuan and 6682.2 yuan in Beijing and Chengdu, respectively (see Table 1), most of those whose income is unstable choose to give up, as one informant explained:

“*Of course, I would like to have social insurance; however, it is flexible and variable for food delivery work, and the income is also unsteady. Sometimes, it is not enough for me to pay my children’s tuition and my own rent. I really can’t afford the entire contribution to social security without any help from platforms or employers*”.

Second, even if platform workers are legally classified as employees, they may not have access to social protection due to the temporary nature of their activities. It is important to understand the difference between statutory access to social protections and effective entitlement. In other words, even though platform workers have a legal right to a social protection (such as endowment insurance), they may not have access in practice, due to non-compliance with specific practical requirements, such as continuity of employment, length of employment, income below a required threshold, intermittent contributions, and so on [23]. For instance, in China, individuals participating in the basic endowment insurance receive a monthly basic pension provided they have contributed premiums for a continuous period of 15 years or more upon reaching the statutory retirement age (60 for men and 55 for women). People who want to receive unemployment insurance benefits must pay the premium for at least one year with their employers. Therefore, fragmented working careers represent an obstacle to building up adequate benefits as part of contribution-based social protection systems. As one platform manager described, the turn-over rate in the take-away industry is extremely high, and many people working as food delivery providers are in a transition period in which they cannot find a permanent job. Therefore, it is too expensive and difficult to purchase social insurance for these workers.

The many different business models, along with flexible and diverse forms of employment in the platform economy, make it difficult to apply a universal approach as in the past. The existing framework for much of public policy–from minimum wages to benefits and pensions–is built around and delivered through the traditionally organized business firm. Digital platforms, operating in the platform economy, follow many different business models and do not sit comfortably with this conventional mode of policy delivery [1]. Meanwhile, the increasing diversity, complexity and scale of the unconventional working arrangements that platforms facilitate raise a series of particularly difficult questions related to existing employment laws. Moreover, platforms and their activities are vastly heterogeneous, in terms of the way they are organized, the sectors they affect/belong to, their size and scope, the type of users they target, etc., making it difficult to apply a one-size-fits-all approach [19]. As one policy maker explained:

“*Since traditional production methods, labor forms and organizational forms are relatively fixed, and labor relations are relatively clear, the social security system established on this basis can operate stably, stipulating that the employer individually or employer and employee jointly bear the responsibility of payment, and withhold or remit through the enterprise. However, platform employment has completely broken the previous pattern, and the existing social security system is simply unable to adapt to the diversified economic model*”.

**Figure 1:**
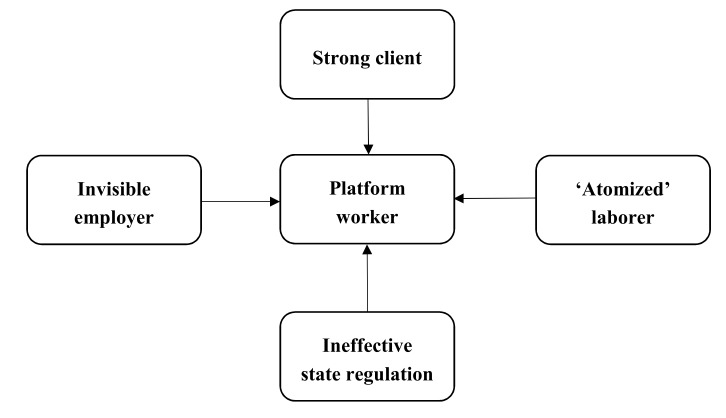
Workers embody an extremely weak position among stakeholders in the digital economy.

Therefore, based on the characteristics of platforms and the peculiarities of platform workers’ employment, we find that these workers embody the most vulnerable position among stakeholders in the digital economy (see Figure 1). The platform, which should play the role of employer, is “hidden”, classifying platform workers as self-employed or independent contractors, to avoid obligations regarding labor rights and the payment of social security contributions. The client-led rating system working as a new dominant “medium” shapes a “strong client”, who can directly impact the income or even the future job prospects of platform workers. Isolated and “atomized” platform workers do not have a fixed workplace, always competing with each other, so they cannot organize an effective union to safeguard their own labor rights. Finally, new regulatory loopholes in the digital space lead to the vast majority of platform workers being excluded from labor protections available to other workers, and they face numerous occupational safety and health risks brought about by platform work.

It is worth mentioning that the issue of new employment and gig employment and the social security of digital employees have also been addressed by some exploratory and tentative studies inside China. For example, the China Association of Employment Promotion (CAEP) published a report in 2014 entitled, “Report on Statistics of Internet Entrepreneurship and Internet Employment and Social Security Research”. This report investigated employment in online shops in China and found that 42 percent of individual online shop owners in China did not participate in any social insurance, and 32.7 percent of online shops owned by enterprises did not participate in any social insurance. Moreover, 75.6 percent of personal online store employees had not participated in any social insurance program, and more than 50 percent of employees in online stores owned by enterprises had not participated in any social insurance program [36]. The trend demonstrated in this report has bear witness to the urgency of labor and social protection within new economy employment.

## 5. Conclusions

Digitalization and the rapid diffusion of digital employment forms under the flagship of the new economy and the indefinite possibilities they bring have indeed shaped and modified the institutional arrangements and power configuration of the labor process. The digital age has shown the potential to redefine the employment relationship and the traditional industrial-labor relationship in industrial societies. Our empirical survey in the Chinese context has traced new trends in digital employment activities, as compared to the brick-and-mortar economy. One of the biggest changes and challenges is related to the concealment of employers in the digital labor market. The initial starting point for the employment relationship emanates from two conflicting, competing and (later) collaborating parties—the capitalist and the working class; in the modern context, they are the employers and the employees. Among the activities conducted over platforms, employers have become implicit, intangible and even “invisible”. The hidden status and indistinctness of digital employers have hollowed out the core of the modern employment relationship. All responsibilities, obligations and duties from the side of employers cannot be fulfilled due to the missing responsibility and liability subjects, since many programs of labor protection and social protection are closely linked to employer responsibilities. Since various internet platforms usually disclaim their status as employers and only define them as information-sharing providers or service providers, millions of platform employees encounter a dilemma. What is hidden are not only the employers, but also the guarantors of social obligation and social responsibility. In the case of infringement upon the labor rights and social rights of new economy employees, it is also difficult to identify the ultimately responsible subject in the legal sense. Even though they claim that they are not employers, network platforms have actually exercised social control over platform employees through adopting rigorous evaluation methods such as scoring and rating systems, and further, through collecting referral fees from platform employees. The network platforms have cunningly hidden their social attribution as holders of quasi-absolute power and have in fact exercised dominant and hegemonic power over platform employees; however, they avoid bearing their responsibilities as employers. In this refined new power configuration of the emerging digital market, the network platforms, as implicit employers, can maximize their economic interest while minimizing their social obligations. The forms of social control over new economy employees have become hidden, refined and sophisticated. Simultaneously, the digital employees have become a group of disenfranchised individuals who lack unions to represent them.

With regard to the interrelationship between platform employees and clients, our survey has further found that an asymmetrical power distribution between both sides has emerged in favor of the unilateral “evaluation power” of clients, which leaves little space for new economy employees to appeal against these unilateral judgements of clients. This power asymmetry has further weakened the position of these employees in the digital labor market, among the newly formed triangle between network platforms, employees and clients. The employees are situated in the least favorable position, structurally disadvantaged in this triangular relationship. The pervasive use of digital communication patterns, including smart phones, apps and software, has tremendously facilitated the power control of network platforms and clients over the quality of digital employment through coercion, penalty and perpetuating service quality evaluation. Meanwhile, employees in digital sectors must bear various social risks, such as road accidents, occupational diseases, physical and psychological stress and pressure. In the epoch of industrial capitalism, a record-keeping system of punch in and punch out had been the control over employees by firms; however, in digital capitalism, control comes from clients typing their evaluation on a keyboard and from online platforms blocking the accounts of e-employees.

The additional gain of power by network platforms and clients corresponds to a substantial loss of power for the digital working class. The organization and mobilization of platform employees have declined to a minimum. Employees are mostly over-atomized and over-individualized without connection to a physical space where workers could meet and interact and thus, forge a natural class consciousness and belongingness. In addition, the comprehensive established social safety network in China has been overshadowed by structural holes and loopholes; platform employees are not covered by social insurance programs, and even the intention to participate in social insurance programs has been remarkably weakened. With the deepening and widening of the digital tide in the Chinese labor market, the social insurance loopholes have become too large to be ignored. A main challenge posed by the new volatile and fragile employment forms communicated through digital devices and software programs is the fundamental disruption of the principle of co-financing social insurance premiums by employers and employees. Since many of these online employees do not have definite employers, the payment of social insurance premiums can only be undertaken by employees themselves, undermining the Bismarck model of social insurance that assumes equal payment of contributions by both sides. The transformation of social policy in the digital age requires a new type of digital governance at the national and local levels to cope with increasing social and public risks [37,38].

The fragile employment relationship and the disrupted labor protection in the new economy have demonstrated that some characteristics of factory despotic regimes [8] may be resurging in the epoch of digitalization, with disempowered, disorganized, disenfranchised and atomized “digital individuals” and cutthroat job competition on the one side and dramatically tightened social control of “digital employees” through the rigorous discipline determined by platforms and clients on the other side. All institutional arrangements in the modern welfare states begin to be softened and weakened in the tide of digital capitalism. This new development calls for a new “counter-movement” to re-strengthen the power of the state to regulate and supervise this highly volatile, intangible and fragile sector, in order to reestablish a digital environment and framework for various employment forms in the new economy.

## Figures and Tables

**Table 1:** Characteristics of platform worker participants in Beijing and Chengdu (*N* = 46).

Characteristics	Beijing (Frequency/Mean)	Chengdu (Frequency/Mean)	All (Frequency/Mean)
City	25	21	46
Age			
Mean (year)	32.6	34.3	33.4
Education background			
Primary school	5	4	9
Junior high school	10	8	18
Senior high school	7	8	15
Higher education	3	1	4
Occupation			
Food delivery worker	13	11	24
Ride-hailing driver	12	10	22
Domicile			
This city	1	5	6
This province	0	10	10
Other province	24	6	30
Labor contract			
Yes	4	2	6
No	21	19	40
Net income			
Mean (yuan)	7720.5	6682.2	7246.5

Source: Authors’ own compilation.
